# Co_0.9_Co_0.1_S Nanorods with an Internal Electric Field and Photothermal Effect Synergistically for Boosting Photocatalytic H_2_ Evolution

**DOI:** 10.3390/ijms23179756

**Published:** 2022-08-28

**Authors:** Lilei Zhang, Manzhou Hong, Ka Zhang, Botan Li, Haipeng Fang, Xun Feng, Xiuchan Xiao

**Affiliations:** 1 Henan Key Laboratory of Function Oriented Porous Materials, College of Chemistry and Chemical Engineering, Luoyang Normal University, Luoyang 471934, China; 2 Green Catalysis Center, College of Chemistry, Zhengzhou University, Zhengzhou 450001, China; 3 School of Materials and Environmental Engineering, Chengdu Technological University, Chengdu 611730, China

**Keywords:** Cd_0.9_Co_0.1_S nanorods, photothermal, internal electric field, pencil-shaped, photocatalytic

## Abstract

The paper reports a strategy to synthesize Cd_0.9_Co_0.1_S nanorods (NRs) via a one-pot solvothermal method. Remarkably, the pencil-shaped Cd_0.9_Co_0.1_S NRs with a large aspect ratio and good polycrystalline plane structure significantly shorten the photogenerated carrier transfer path and achieve fast separation. An appropriate amount of Co addition enhances visible light-harvesting and generates a photothermal effect to improve the surface reaction kinetics and increases the charge transfer rate. Moreover, the internal electric field facilitates the separation and transfer of carriers and effectively impedes their recombination. As a result, the optimized Cd_0.9_Co_0.1_S NRs yield a remarkable H_2_ evolution rate of 8.009 mmol·g^−1^·h^−1^, which is approximately 7.2 times higher than that of pristine CdS. This work improves the photocatalytic hydrogen production rate by tuning and optimizing electronic structures through element addition and using the photothermal synergistic effect.

## 1. Introduction

Hydrogen is essential in the clean energy system [1,2,3,4]. Semiconductor-based photocatalytic hydrogen production is promising to alleviate increasing energy and pollution problems [5,6,7,8,9]. Many metal sulfide-based semiconductors have been discovered for photocatalytic hydrogen evolution. Represented by CdS, exhibiting good visible light response, narrow bandgap, and morphology controllability, it is considered one of the most promising photocatalytic materials. However, because of the severe photo-corrosion and rapid recombination of the photogenerated carrier of pure CdS, its proton-reduction efficiency is limited, resulting in poor photocatalytic performance [10,11,12].

Hence, several researchers have reported various strategies to elevate the photocatalytic performance of CdS-based materials. First of all, CdS with multiple shapes, such as nanoparticles [13], nanotubes [14], nanospheres [15], and nanorods [16] have been found in many studies and showed excellent photocatalytic performance. Among them, the CdS nanorods have attracted more and more attention because of their large aspect ratio and fewer defects, which can significantly shorten the radial transfer path of carriers and achieve rapid charge separation, thus exhibiting high photocatalytic hydrogen evolution performance [17,18]. In addition, several other methods have been applied, such as constructing an intrinsic built-in electric field, defect and heterostructure engineering, and co-catalyst decoration to accelerate the separation and transfer of photogenerated electron-hole pairs [19,20,21,22]. Establishing the internal electric field in semiconductors by element doping can promote the separation and transfer of photogenerated electrons, and extract carriers, thus improving the photocatalytic efficiency. For example, the Huang group prepared a gradient P-doped CdS nanostructure via surface diffusion doping strategy [23] the Hu group reported gradient-P-doped CdS/CoP hybrid nanorods [24], and the Shangguan group explored Ni, Mo, Co-doped CdS for efficient photocatalytic hydrogen evolution from water splitting [25].

It is well known that photothermal conversion is an attractive strategy for solar light-harvesting, and the local temperature can go up in situ, which generally enhances the entropy of chemical reactions and enhances the kinetics of surface catalytic reactions to accelerate the transfer of photogenerated charges, thus increasing the photocatalytic reaction rate [26,27,28,29,30]. Especially in water splitting that involves a brutal thermodynamic uphill reaction, heat as an external force can effectively drive water photocatalytic hydrogen production [31]. The photothermal synergistic catalytic system that couples photochemical and thermochemical effects shows better performance than the traditional single catalytic system [32]. Therefore, using the photothermal effect to enhance CdS-based performance should be an excellent strategy.

**Scheme 1:**
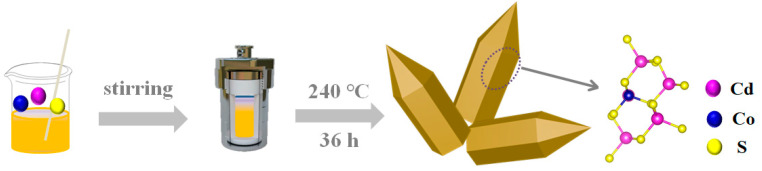
Schematic illustration of the synthetic process for Cd_0.9_Co_0.1_S nanorods structure.

Herein, pencil-tipped CdCoS NRs were prepared by a one-pot method, as presented in Scheme 1. In this work, we added an appropriate amount of Co into CdS NRs, which narrowed the pure CdS bandgap and significantly enhanced light-harvesting ability, achieving rapid charge separation efficiency. Meanwhile, element addition realized the construction of the internal electric field and the photothermal synergistic. Finally, the optimized Cd_0.9_Co_0.1_S NRs photocatalyst exhibits excellent hydrogen production efficiency under visible light illumination.

## 2. Results and Discussion

### 2.1. Structure and Morphology Analysis of Photocatalysts

**Figure 1:**
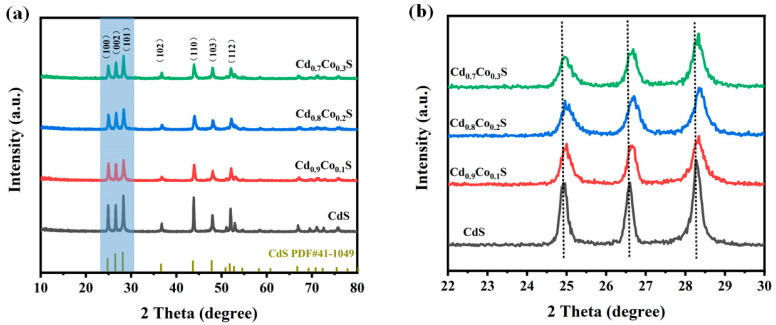
The XRD patterns (**a**) and partially magnified XRD patterns of pure CdS and CdCoS NRs (**b**).

XRD analyzed the phase and crystal structure of the synthesized samples. As presented in Figure 1a, hexagonal phase CdS existed in all samples. The XRD pattern of pure CdS displayed several obvious peaks at 24.8, 26.5, 28.1, 36.6, 43.6, 47.8, and 51.8°, attributed to the (100), (002), (101), (102), (110), (103), and (112) planes, respectively (PFD#41-1049) [16,33]. After Co addition, the diffraction peak shifted of Cd_0.9_Co_0.1_S NRs relative to pure CdS, and there was no obvious peak of cobalt sulfide present, indicating the change of its crystal structure. Specifically, the partially magnified XRD pattern of samples showed that the main diffraction peak positions on the CdCoS samples were slightly shifted to a higher 2θ value, which means that the lattice parameter changes [34]. The shift can be attributed to the successful incorporation of Co^2+^ into CdS because the effective ionic radius of Co^2+^ is smaller than that of Cd^2+^ [35,36]. Furthermore, the contents of Cd, Co, and S of the obtained CdCoS photocatalysts were further determined by ICP-MS. As presented in Table S1, the molar ratios of Co/Cd were 0.103, 0.228, and 0.319, corresponding to the samples Cd_0.9_Co_0.1_S, Cd_0.8_Co_0.2_S, and Cd_0.7_Co_0.3_S, respectively.

**Figure 2:**
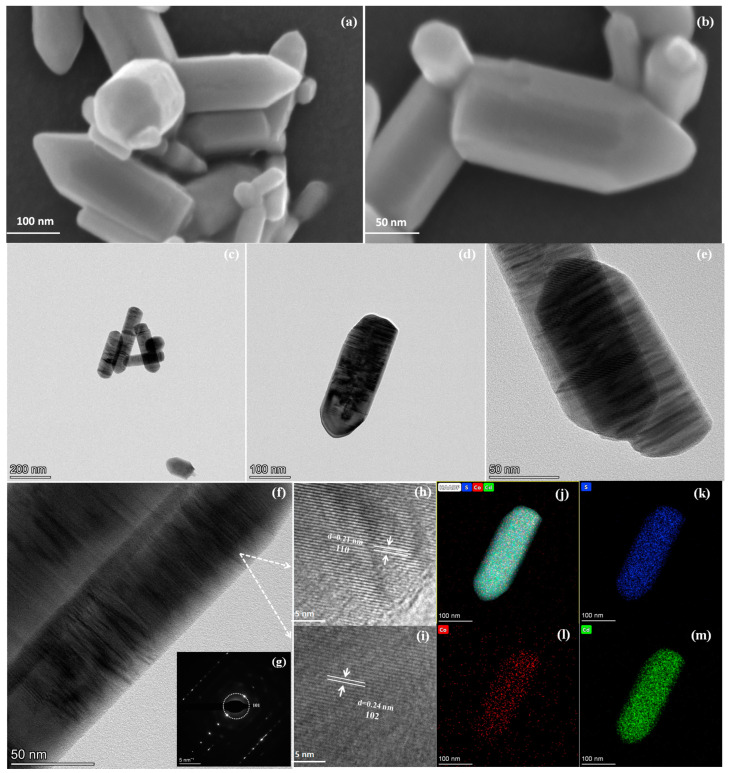
SEM images (**a**,**b**) and TEM images (**c**–**f**), SAED pattern (**g**), HRTEM images (**h**,**i**), and elemental mappings (**j**–**m**) of the Cd_0.9_Co_0.1_S NRs.

The SEM and TEM images give detailed information on the microscopic structures and morphologies of Cd_0.9_Co_0.1_S NRs. As the SEM images (Figure 2a,b) revealed, Cd_0.9_Co_0.1_S NRs displayed a clear crystal structure with a regular polyhedral shape. The TEM images (Figure 2c–f) of Cd_0.9_Co_0.1_S showed the nanorod-like system. It can be seen that the crystal surface is smooth after Co addition, and no obvious Co nanoparticles are found. Meanwhile, as the SAED pattern (Figure 2g) and HRTEM images (Figure 2h,i) presented, the corresponding lattice diffraction pattern of Cd_0.9_Co_0.1_S NRs with equal lattice diffraction spacing was well matched with the (101) plane of the CdS phase. The lattice fringes with interlayer distances of 0.21 and 0.24 nm were assigned to the (110) and (102) planes [37], respectively, of pristine CdS (PDF#41-1049). In addition, elemental mapping results (Figure 2j–m) and energy dispersive spectroscopy (EDS) spectra (Figure S1) of an individual Cd_0.9_Co_0.1_S NRs sample further confirm the existence of S, Cd, and Co elements in the photocatalyst, indicating the Co has been added successfully. 

**Figure 3:**
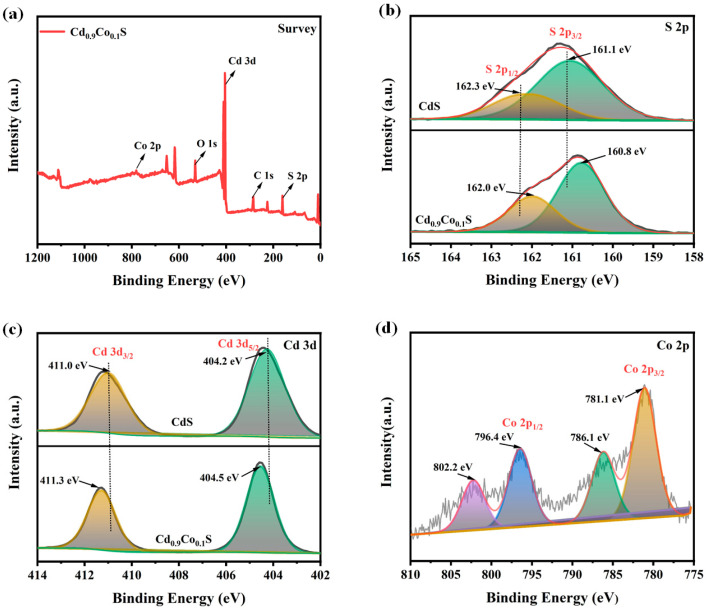
XPS survey spectra of Cd_0.9_Co_0.1_S NRs (**a**), and high-resolution XPS spectra of: S 2p (**b**), Cd 3d (**c**) in the CdS and Cd_0.9_Co_0.1_S NRs, and Co 2p (**d**) in the Cd_0.9_Co_0.1_S NRs.

XPS studied the surface chemical state and internal elemental bond composition of samples. Figure 3a displayed the full-survey spectrum of Cd_0.9_Co_0.1_S NRs, indicating the existence of Cd, S, C, O, and Co elements. The S 2p spectrum of Cd_0.9_Co_0.1_S NRs in Figure 3b showed two binding energies at 160.8 and 162.0 eV, ascribed to S 2p_3/2_ and S 2p_1/2_, respectively. Simultaneously, as presented in Figure 3c, the Cd 3d spectrum of Cd_0.9_Co_0.1_S NRs can be deconvoluted into Cd 3d_5/2_ (404.5 eV) and Cd 3d_3/2_ (411.3 eV) [31,38]. Significantly, compared with pure CdS, the two peaks of S 2p in Cd_0.9_Co_0.1_S NRs shifted to the low binding energy region. Conversely, the characteristic peaks of Cd 3d shifted slightly to a higher position, which indicates that a fraction of Co atoms has substituted Cd sites to form the Co-S bond, resulting in the redistribution of charge around Cd and S atoms [37,39]. Moreover, the Co 2p spectrum (Figure 3d) of Cd_0.9_Co_0.1_S NRs can be deconvoluted into four peaks. The peaks located at 781.1 and 786.1 eV and at 796.4 and 802.2 eV corresponded to Co 2p_3/2_ and Co 2p_1/2_, respectively [40,41]. Such results further indicated that Co had been successfully added into the composites.

### 2.2. Photocatalytic H_2_ Evolution Performance

**Figure 4:**
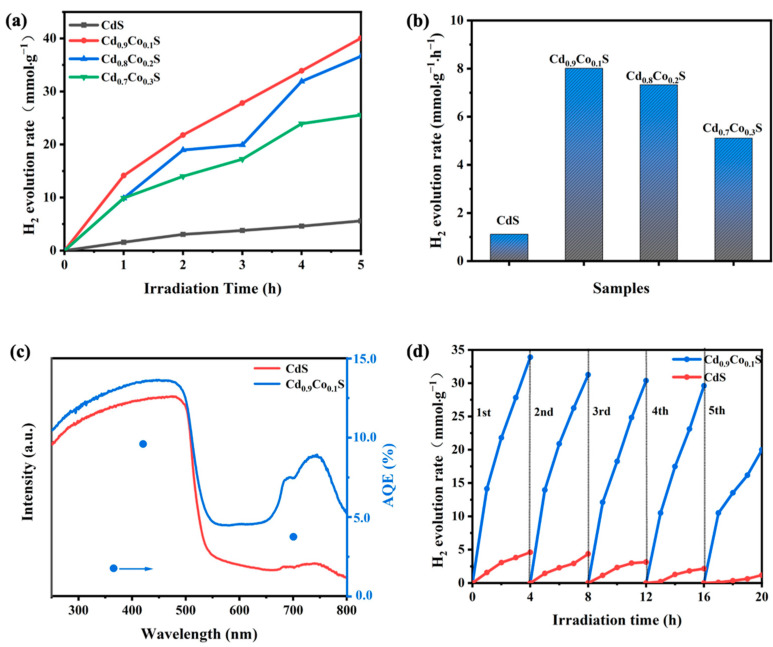
Time-dependent photocatalytic H_2_ evolution (**a**) and H_2_ evolution rates for all samples (**b**). Apparent quantum efficiency (AQE) and the UV–vis absorption spectrum of Cd_0.9_Co_0.1_S NRs (**c**). The AQE equals twice the number of evolved H_2_ molecules divided by the number of incident photons under light irradiation with specific wavelengths (365, 420, and 700 nm). Cyclic tests of photocatalytic H_2_ evolution for pure CdS and Cd_0.9_Co_0.1_S (**d**).

Photocatalytic H_2_ evolution performance was evaluated in the prepared samples. As demonstrated in Figure 4a,b, pure CdS exhibited the lowest H_2_ evolution rate of 1.12 mmol·g^−1^·h^−1^, gradually declining in the reaction. After Co addition, the H_2_ evolution rate of all CdCoS NRs was significantly enhanced. In particular, the hydrogen evolution rate of Cd_0.9_Co_0.1_S NRs reached the highest value of 8.009 mmol·g^−1^·h^−1^ (Video S1), which is about 7.2 times that of pure CdS. It surpassed most of the other CdS-based photocatalysts previously reported (Table S2). With the increase of Co addition, the hydrogen evolution rate gradually reduced because excessive added elements may become new carrier recombination centers, which hindered the proton-reduction process [42]. Therefore, the appropriate amount of Co addition is essential to enhance photocatalytic activity significantly. The apparent quantum efficiency (AQE) is an important measure of photocatalytic hydrogen production performance, equal to twice the number of evolved H_2_ molecules divided by the number of incident photons. The AQE of Cd_0.9_Co_0.1_S NRs under different monochromatic light irradiations were calculated. As shown in Figure 4c, it was 1.77, 9.60, and 3.80% at 365, 420, and 700 nm, respectively, which indicated that the photocatalyst had a modest utilization efficiency of light. It confirmed the crucial role of the photothermal synergistic effect in significantly improving the H_2_ generation performance of Cd_0.9_Co_0.1_S NRs.

Moreover, the photocatalytic H_2_ production stability of Cd_0.9_Co_0.1_S NRs was evaluated through a 20 h cyclic photocatalytic test, with a total of five cycles, each cycle of 4 h, and the results were shown in Figure 4d. In the first three cycles, the hydrogen production of the sample remained unchanged. In the 5th cycle reaction, the hydrogen evolution rate of Cd_0.9_Co_0.1_S NRs has decreased. However, it was still significantly higher than pure CdS, possibly due to the loss of photocatalyst quality and sacrificial agent in the reaction process. Therefore, we supplemented the sacrificial agent and photocatalyst after five cycles of stability testing. The results (Figure S4a) showed that the hydrogen production performance increased after the supplement but decreased compared to the initial examination. 

It is known that the photo-corrosion of CdS is one of the main reasons affecting its performance. When the CdS is photo-corroded, the Cd^2+^ will be dispersed into the dispersion solution. To clarify the degree of photo-corrosion of the photocatalyst during the reaction, the Cd^2+^ concentration in the reaction solution of each run was monitored by ICP. As displayed in Figure S4b, the Cd^2+^ concentration in pure CdS increases rapidly with the progress of the reaction. Meanwhile, it can be seen from the XPS spectra (Figure S5) after the cyclic response that there is a small amount of S^0^ in the S 2p spectrum of pure CdS, which was attributed to the oxidation of divalent sulfur, indicating that obvious photo-corrosion occurred in pure CdS during the reaction process.

In comparison, the Cd^2+^ concentration in Cd_0.9_Co_0.1_S NRs has increased but was much lower than pure CdS. This result showed that Cd_0.9_Co_0.1_S NRs have a little photo-corrosion under long-time illumination, leading to its performance decline. However, compared with pure CdS, Co addition reduced photo-corrosion and improved the stability of the photocatalyst. Furthermore, the SEM images (Figure S2) and XRD spectra (Figure S6) of Cd_0.9_Co_0.1_S NRs showed that the morphology and phase of the photocatalyst did not change after cyclic tests, indicating that the photocatalyst had good stability.

### 2.3. Photothermal Effect

**Figure 5:**
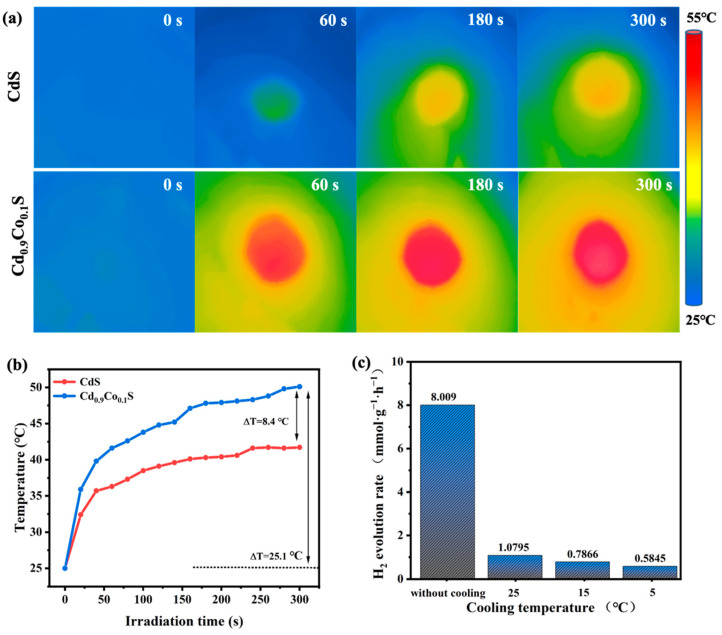
Infrared thermography photos (**a**), the time-dependent temperature of the pure CdS and Cd_0.9_Co_0.1_S NRs under visible light (**b**), and H_2_ evolution rates of Cd_0.9_Co_0.1_S NRs without and with cooling the reaction solutions (**c**).

Temperature is one of the influential critical factors in photocatalytic reaction, and an appropriate temperature increase is conducive to photo-catalytic response [43,44]. To verify the photothermal synergistic effect of the photocatalytic, the surface temperature changes of pristine CdS and Cd_0.9_Co_0.1_S NRs under visible light irradiation were tracked in situ. As illustrated in Figure 5a,b, the surface temperatures of Cd_0.9_Co_0.1_S NRs increased rapidly in 300 s and reached 50.1 °C from 25.0 °C, which was higher than that of pristine CdS. Compared with pure CdS, the Cd_0.9_Co_0.1_S NRs exhibited better photothermal properties, indicating that the Co-S bond formed after proper Co addition played a crucial role in the photothermal effect of photocatalytic reaction [45,46]. In the photothermal phenomenon, as the conversion from light to heat, high temperature can generally accelerate the transfer of charge carriers and surface reaction kinetics, thus improving the photocatalytic performance [47,48,49]. To investigate the effect of temperature on photocatalytic H_2_ generation, the photocatalytic H_2_ evolution rate of Cd_0.9_Co_0.1_S NRs at different temperatures was measured. As shown in Figure 5c, the H_2_ evolution rate declined sharply with the decrease in reaction temperature, indicating that the excellent photothermal effect of Cd_0.9_Co_0.1_S NRs played an essential role in its better photocatalytic performance. 

### 2.4. Optical Property and Band Structure

**Figure 6:**
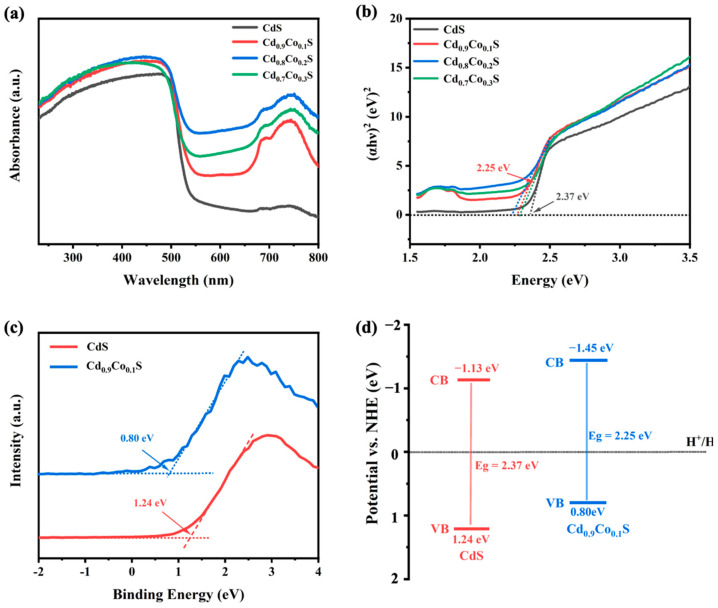
UV–vis diffuse reflectance spectra (**a**), corresponding Tauc plots (**b**), XPS valence band spectra (**c**), and Schematic band structures of pure CdS and Cd_0.9_Co_0.1_S NRs (**d**).

The UV-vis absorption spectra of the samples were measured to analyze their light-absorption properties. As displayed in Figure 6a, the pure CdS has an inherent bandgap (Eg) adsorption with a band edge of about 520 nm. Compared with pure CdS, with the increase of Co addition, the CdCoS NRs exhibited better light absorption capacity in the wavelength range of 230–520 nm, and all CdS-based photocatalysts had a redshift. Notably, CdCoS NRs showed an obvious tail absorbance in the field of 550–800 nm. It speculates that the tail absorbance in the 550–800 nm field is mainly caused by the vibration of Co atoms in the lattice. As the band gap of Cd_0.7_Co_0.3_S NRs became wider (Figure 6b), the absorbance decreased slightly. However, it was still significantly higher than pure CdS, demonstrating that Co addition can enhance the light-harvesting ability of the photocatalysts. Meanwhile, the samples showed a strong absorption peak around 740 nm, which may enable them to have a higher photothermal conversion efficiency.

The appropriate band structure of the catalyst is essential to charge-oriented transfer, with a suitable bandgap, the suitable conduction band (CB) and valance band (VB) edge potentials [50]. As displayed in Figure 6b, the bandgap energy of CdS and Cd_0.9_Co_0.1_S NRs calculated based on the Kubelka–Munk method from the Tauc plots were 2.37 and 2.25 eV, respectively. As presented in Figure 6c, the VB value of pure CdS and Cd_0.9_Co_0.1_S NRs were 1.24 and 0.80 eV, respectively. The VB value is the energy of the intersection point obtained by extending the straight part near 0 eV and extending the horizontal part less than 0 eV. Moreover, the Mott-Schottky method measured the E_fb_ (flat-band potential) of pristine CdS and Cd_0.9_Co_0.1_S NRs. As shown in Figure S3a,b, the Mott–Schottky plots of CdS and Cd_0.9_Co_0.1_S NRs showed a positive slope, indicating that semiconductors have n-type characteristics [51], and the flat band potentials (E_fb_) of CdS and Cd_0.9_Co_0.1_S NRs were approximately −1.17 and −1.49 V (vs. SCE), respectively. The measured potentials can be converted to the standard hydrogen electrode (NHE) scale by the Nernst equation [52] (Equation (1)):

E_(NHE)_ = E_(SCE)_ + 0.24 (1)

By calculation, the E_fb_ of CdS and Cd_0.9_Co_0.1_S NRs were −0.93 and −1.25 V (vs. NHE), respectively. Theoretically, the conduction band position of n-type semiconductors is −0.2 V negative than flat band potential [39,53]. So, the conduction band positions (E_CB_) of CdS and Cd_0.9_Co_0.1_S NRs were −1.13 and −1.45 eV, respectively. The formula E_g_ = E_VB_ − E_CB_ shows that the calculated results match well with the test results. Therefore, the band energy alignment of CdS and Cd_0.9_Co_0.1_S NRs can be determined (Figure 6d). It can be seen that Co addition can narrow the bandgap, and the Cd_0.9_Co_0.1_S NRs have a more negative CB, indicating that it has a higher reduction power and is more beneficial to solar hydrogen production [50,54].

### 2.5. Separation and Transfer of Charge Carriers

**Figure 7:**
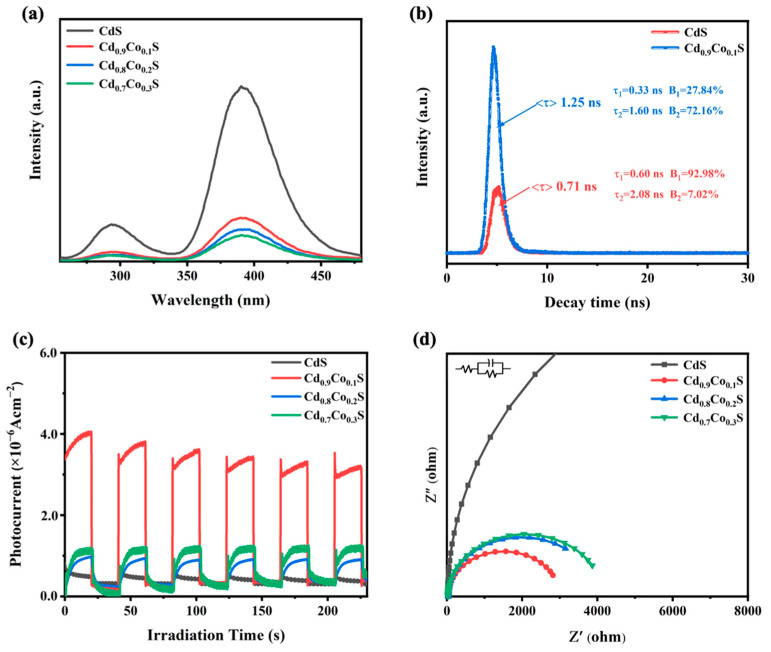
PL spectra (**a**), TRPL decay spectra (**b**), photocurrent responses (**c**), and EIS plots (**d**) of as-prepared samples.

A series of photoelectron-chemical measurements were carried out to discuss further the reasons for the excellent photocatalytic performance of Cd_0.9_Co_0.1_S NRs. Generally speaking, the photoluminescence (PL) peak’s low intensity indicates the charge carriers’ low recombination efficiency [55]. It can be seen from Figure 7a that the pure CdS showed the strongest PL peak at about 390 nm, which is owing to the rapid recombination of electrons and holes. After Co addition, the P.L. signal of the sample was significantly reduced, which indicated that Co addition could effectively accelerate the charge separation and reduce its recombination efficiency. However, with the increase of Co addition, too much Co formed new active sites, further reducing the recombination of photogenerated carriers, so the sample Cd_0.7_Co_0.3_S NRs had the lowest PL signal. In addition, the time-resolved photoluminescence (TRPL) spectra showed the average lifetime of charge carriers of samples under irradiation, and the results are illustrated in Figure 7b and S7. The average lifetimes of carriers were 0.71, 1.25, 0.96, and 0.89 ns for pure CdS, Cd_0.9_Co_0.1_S, Cd_0.8_Co_0.2_S, and Cd_0.7_Co_0.3_S NRs, respectively. The Cd_0.9_Co_0.1_S NRs exhibited the longest carrier lifetime, indicating that the Co addition in Cd_0.9_Co_0.1_S can significantly improve electron-hole pairs separation efficiency [56], and a long lifetime of the carrier means more opportunities to participate in the hydrogen evolution reactions [57]. The above results indicated that Co addition could effectively capture electron-hole pairs and thus effectively prevent their recombination.

Transient photocurrent tests examined the efficiency of separating the electron-hole pairs. As presented in Figure 7c, Cd_0.9_Co_0.1_S NRs showed the highest photocurrent density among all samples, indicating the highest charge separation efficiency. Compared with pristine CdS, an appropriate amount of Co addition can accelerate the separation and transfer of charge carriers. Furthermore, electronic transmission capacity is a significant factor for catalysts, and electrochemical impedance spectroscopy (EIS) was utilized to study the resistance of the electrode/electrolyte interface of different catalysts during the photocatalytic process [58]. As can be seen clearly from Figure 7d, the radius of the Nyquist circle of the Cd_0.9_Co_0.1_S NRs was significantly smaller than that of other samples, indicating that it has the minor charge transfer resistance to ensure the fastest charge transfer rate. In summary, the above photochemical characterization results confirm that the Cd_0.9_Co_0.1_S NRs can improve charge separation, enhance charge transfer and prolong charge lifetime, thus significantly enhancing the photocatalytic performance of hydrogen evolution.

### 2.6. Photocatalytic Mechanism

**Figure 8:**
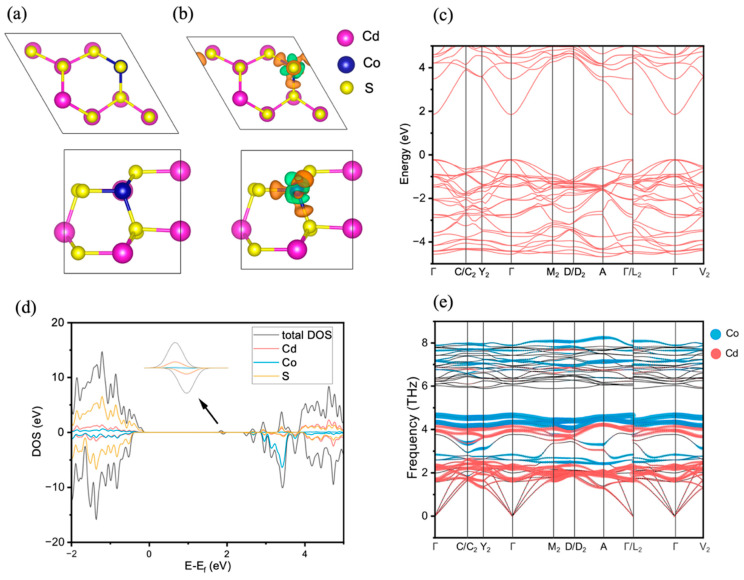
DFT calculation: side views of structural model (**a**), and Charge density differences of Cd_0.9_Co_0.1_S NRs (**b**) brown (gain electron), green (lose electron), Electronic band structure (**c**), and Total and projected density of states for Cd_0.9_Co_0.1_S NRs (**d**) (spin-up is upper half, spin-down is lower half), and Phonon spectra for Cd_0.9_Co_0.1_S NRs (**e**).

To deeply understand the electronic regulation effect of Co addition strategy in CdS, density functional theory (DFT) calculations were performed. As shown in Figure 8a, the structure model of Cd_0.9_Co_0.1_S NRs was constructed. After Co addition, the local electronic structure of Cd_0.9_Co_0.1_S NRs was changed. Figure 8b,c showed the optimal configuration and different charge densities, and the charge of Cd_0.9_Co_0.1_S NRs also depleted or accumulated in other regions. Specifically, Co atoms caused the loss of surrounding electrons. Meanwhile, the electron density of S atoms increased (the brown and green isosurfaces depict electron accumulation and electron depletion, respectively). Therefore, an internal electric field was built in Cd_0.9_Co_0.1_S NRs, which boosted the separation of electrons and holes, and inhibited its recombination [25].

Based on the frontier molecular orbital theory, the region near the Fermi level is essential for photocatalysis [59,60]. As shown in Figure 8c, with Co addition, the bandgap of Cd_0.9_Co_0.1_S NRs 2.05 eV was narrower than that of pure CdS 2.12 eV (Figure S8), which helps the absorption of visible light. The simulation results were consistent with the experimental value. The valence band maximum (VBM) and conduction band minimum (CBM) of Cd_0.9_Co_0.1_S NRs are located at the same Γ point, indicating that the sample is a direct bandgap semiconductor [49,61]. It is noteworthy that metallic materials can generate electrons and holes through inter-band and intra-band transitions [62]. Therefore, the sample can produce electron-hole pairs more easily under visible light irradiation. Furthermore, the density of states (DOS) and partial density of states (PDOS) of Cd_0.9_Co_0.1_S NRs were displayed in Figure 8d. The partial PDOS of Cd_0.9_Co_0.1_S NRs was decomposed according to the contribution of different elements. As a result, the VBM is principally contributed by S orbital, and the CBM is mainly contributed by the hybridization of S, Cd, and Co orbitals, thus forming the Co-S bond. After absorbing photons, the Co-S bonds can act as a “bridge” for charge transfer, thus inhibiting the carrier recombination [38]. Figure 8e shows the phonon spectrum of Cd_0.9_Co_0.1_S NRs and the contribution of Co and Cd atoms. It can be seen that the Co addition generates new lattice vibration frequencies, which will lead to solar light absorptions. It is consistent with the characterization results of UV-Vis. In addition, the high-frequency vibration is mainly due to the contribution of Co atoms, which will lead to a strong thermal effect, thus increasing the emission of hydrogen and the reaction rate of hydrogen production.

**Scheme 2:**
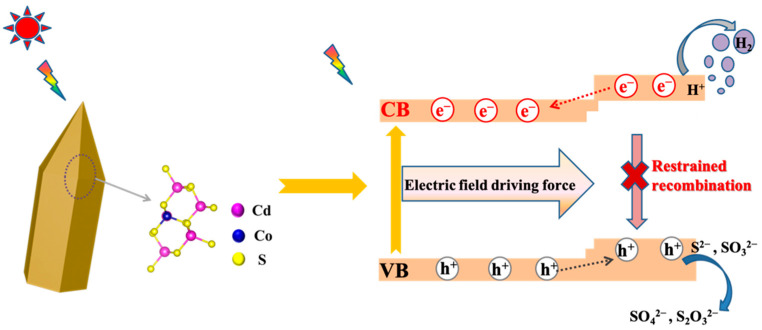
Diagram of the proposed photocatalytic mechanism of Cd_0.9_Co_0.1_S NRs under visible light irradiation.

Based on the above analysis, the possible mechanism for the charge carrier excitation and migration processes of Cd_0.9_Co_0.1_S NRs photocatalyst can be depicted in Scheme 2. Firstly, Co^2+^ partially filled with d orbital generates electron donor levels in the bandgap when it partially enters the CdS lattice, thus narrowing the bandgap. Under visible light irradiation, the narrower bandgap of Cd_0.9_Co_0.1_S NRs is more conducive to light-harvesting and generating electrons and holes. Then, Co addition caused spontaneous electron density redistribution in Cd_0.9_Co_0_.1S NRs. In detail, Co atoms caused the loss of surrounding electrons, and the electron density of S atoms increased.

Meanwhile, the E_VB_ and E_fb_ of Cd_0.9_Co_0.1_S NRs rose, and the band gap decreased. After illumination, due to the generation and migration of carriers, E_fb_ equilibrates and band bends because of the generation and migration of carriers, thus forming an internal electric field. The formed internal electric field can significantly inhibit the recombination of electron-hole pairs and prolong the lifetime of the charge carrier. In addition, the extra energy is converted from chemical energy to thermal energy through photothermal reaction under light irradiation, thereby accelerating the migration rate of carriers in Cd_0.9_Co_0.1_S NRs. Eventually, the electrons gathered in the CB of Cd_0.9_Co_0.1_S NRs encounter H^+^ and release H_2_. The holes accumulated in the VB can obtain enough power to oxidate the sacrificial agent. 

## 3. Materials and Methods

### 3.1. Characterization

The morphologies were determined by SEM (Tecnai G20) and TEM (FEI Talos F200S). XRD patterns were analyzed using a Bruker D8 Advance X-ray diffractometer. The element content of the samples was measured by ICP-MS (PerkinElmer NexION 300X). XPS spectra were conducted using an ESCALAB 250xi spectrometer. FT-IR spectra were recorded on Nicolet 6700 infrared spectrometer. UV-vis diffuse reflectance spectra were recorded on a UH-4150 spectrophotometer, and the bandgap was calculated using the absorption data. Photoluminescence emission spectra were recorded on the Hitachi F-7000 Fluorescence spectrophotometer. The fluorescence lifetime of the sample was measured and exponentially fitted by Edinburgh FLS1000 Photoluminescence Spectrometer. The FLIR monitored infrared thermal images of the sample.

### 3.2. Synthesis of CdCoS NRs

CdCoS NRs were synthesized via a one-pot solvothermal method. In a typical synthesis, firstly, 9 mmol cadmium acetate (Cd(CH_3_COO)_2_·2H_2_O), 12.5 mmol thioacetamide, and 1 mmol cobalt nitrate (Co(NO_3_)_2_·6H_2_O) were dissolved in 15 mL of ethylenediamine and 15 mL of deionized water. Then, the mixture solution was transferred into a 50 mL Teflon-lined stainless-steel autoclave and stirred at room temperature for 1 h. Finally, the mixture solution was maintained at 240 °C for 36 h. After suction filtration and vacuum drying, the obtained sample was simply denoted as Cd_0.9_Co_0.1_S (Cd/Co = 9:1). Meanwhile, under the same experimental conditions, the molar ratio of Cd(CH_3_COO)_2_·2H_2_O and Co(NO_3_)_2_·6H_2_O was changed to 8:2 and 7:3 respectively, and the obtained samples were named as Cd_0.8_Co_0.2_S (Cd/Co = 8:2) and Cd_0.7_Co_0.3_S (Cd/Co = 7:3), respectively.

### 3.3. Photocatalytic H_2_ Evolution

The photocatalytic H_2_ evolution experiment was carried out in a top-irradiated quartz reactor (Labsolar-6A, Perfectlight) with a circulating water system. Typically, 80 mg of photocatalysts were dispersed by ultrasound in a 100 mL Na_2_SO_3_ (0.35 M) and Na_2_S (0.25 M) mixture [63]. Before performing the photocatalytic experiments, the reaction vessel was purged with N_2_ and evacuated for at least 30 min to remove dissolved air. The 300 W Xe-lamp was used as a light source. GC-9790 (Ar as the carrier) gas chromatograph tested the separated hydrogen gas with a thermal conductive detector. Under the same conditions, the blank experiment revealed no appreciable H_2_ evolution without photocatalysts.

### 3.4. The Apparent Quantum Efficiency Analysis

The apparent quantum efficiency (AQE) was performed under the same reaction conditions except using various band-pass filters (365, 420, and 700 nm) to obtain the monochromatic light, and the following equation calculated the value of AQE:

$$AQE=\frac{2\times the\;number\;of\;evolved\;H_{2}\;molecules}{the\;number\;of\;incident\;photons}\times 100\%$$

Details on experimental materials, characterization, photocatalytic H_2_ evolution, and photoelectrochemical measurements are shown in the Supporting Information.

### 3.5. Theoretical Calculation

The Vienna Ab-initio Simulation Package (VASP) [64,65,66] based on the projector augmented wave (PAW) method was carried out for the first principle calculations. All material configurations were geometrically optimized by relaxing the ions and lattice with the Perdew–Burke–Ernzerhof (PBE) parameterization and Monkhorst–Pack k-meshes of 6 × 6 × 8 until the maximum residual ionic force is less than 10^−4^ eV. The cutoff energy of the plane-wave was set to 400 eV. The accurate band structures and density of occupied states were obtained using Heyd–Scuseria–Ernzerhof (HSE06) hybrid density functional [67]. The second interatomic force constants and phono structure were obtained using the Phonopy package [68] with 2 × 2 × 2 supercells. The Vaspkit software package [69] was adopted to analyze the band structures and density of occupied states, and the Vesta software [70] was used to visualize the calculation results.

## 4. Conclusions

In summary, through a one-pot strategy, we successfully prepared Cd_0.9_Co_0.1_S NRs with excellent H_2_ production performance (8.009 mmol·g^−1^·h^−1^). The pencil-shaped Cd_0.9_Co_0.1_S NRs can shorten the radial transfer path of electrons and realize rapid charge separation. Meanwhile, the Co addition significantly enhanced visible light-harvesting, reduced the bandgap, and generated a photothermal synergy to improve the charge transfer rate and surface reaction kinetics. Moreover, the internal electric field accelerates the separation and migration of charge carriers and effectively inhibits the recombination of electrons and holes. This work may provide an optimal platform to design and construct stable nano photocatalysts for solar energy conversion.

## Data Availability

Not applicable.

## Supplementary Materials

The following supporting information can be downloaded at: https://www.mdpi.com/article/10.3390/ijms23179756/s1. References [71,72,73,74,75,76,77,78,79,80,81] are cited in the supplementary materials.
