# Supplementary material for: Co0.9Co0.1S Nanorods with an Internal Electric Field and Photothermal Effect Synergistically for Boosting Photocatalytic H2 Evolution
Source: Int J Mol Sci. 2022 Aug 28;23(17):9756. doi: 10.3390/ijms23179756 (PMC9456290; doi:10.3390/ijms23179756)
Supplement: Supplementary file 1 [file ijms-23-09756-s001.zip › Supplementary Materials.pdf]

Supplementary Materials

$Co_{0.9}Co_{0.1}S$ nanorods with an internal electric field and photothermal effect synergistically for boosting photocatalytic H_2 evolution

1. Experimental section

1.1. Materials

All other reagents used in this research are analytically pure and can be used without further purification. Cadmium diacetate dihydrate ($C_4H_6CdO_4·2H_2O$), Cobaltous nitrate ($Co(NO_3)_2·6H_2O$), Thioacetamide (TAA), Ethylenediamine (EDA) and deionized water.

1.2. Preparation of photocatalysts

1.2.1. Synthesis of CdS NRs

Briefly, 9 mmol $C_4H_6CdO_4·2H_2O$ and 12.5 mmol TAA were dissolved in 15 ml of EDA and 15 ml of deionized water. The resulting solution was transferred to a 50 ml Teflon-lined stainless-steel autoclave, then stirred at room temperature for one hour, and finally maintained at 240 °C for 36

hours. After cooled down to room temperature, the yellow precipitate was collected by suction filtration separation and washed with deionized water and absolute ethanol for several times to remove impurities.

1.3. Photoelectrochemical

Mott-Schottky plots, transient photocurrent response, and electrochemistry impedance spectroscopy (EIS) were measured using CHI660E workstation with a conventional three-electrode system. (the FTO glass electrode was used as working electrode, the platinum foil as counter electrode and the saturated calomel electrode (SCE) as reference electrode). To be specific, a slury prepared by dispersing 5 mg of the sample in 0.5 ml of ethanol and water (4:1) and 20 μl of Nafion solution was spread on FTO glass and used after drying. The light source was a 300W xenon lamp, tested in 0.5M sodium sulfate solution

1.4. Photothermal tests

The photothermal test of as-prepared samples was carried out as follow. 5 mg of the photocatalyst sample was dispersed in 0.5 ml of ethanol and water (4:1) and 20 μl of Nafion solution was spread on a Petri dish and used after drying overnight. The initial temperature was controlled at room temperature and the temperature of the sample was monitored using an

infrared imaging device (FLIR). A 300 W xenon lamp was used as a light source.

2. Supporting data

Table S1 The percentages of Cd, Co, and S elements of $Cd_{0.9}Co_{0.1}S$ $Cd_{0.8}Co_{0.2}S$, and $Cd_{0.7}Co_{0.3}S$ samples

Samples	Element weight content (%)			Co/Cd
	Cd	Co	S	
$Cd_{0.9}Co_{0.1}S$	73.06	7.54	19.54	0.103
$Cd_{0.8}Co_{0.2}S$	59.53	13.55	26.92	0.228
$Cd_{0.8}Co_{0.2}S$	55.06	17.54	27.40	0.319

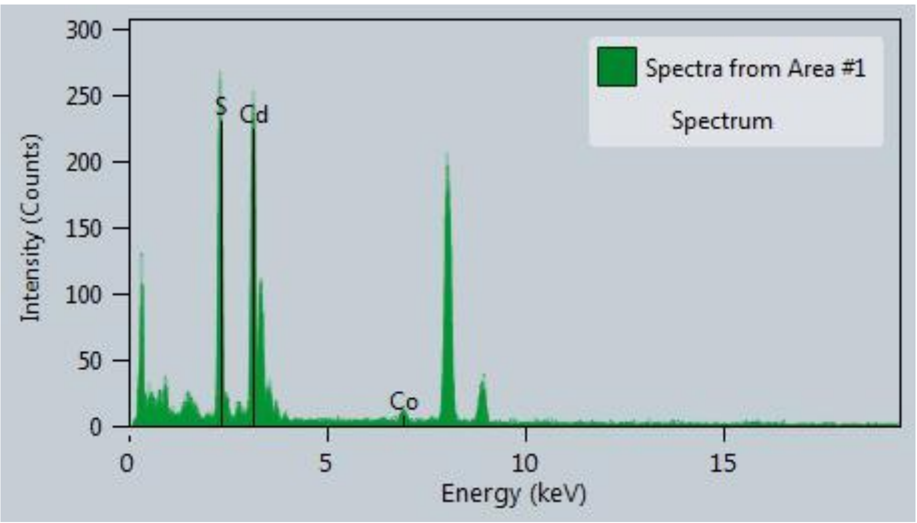

Figure S1 EDS spectra of the sample $Cd_{0.9}Co_{0.1}S$ NRs.

Table S2 Comparison of photocatalytic H_2 evolution rates of different CdS-based photocatalysts.

Photocatalysts	Maximum rate ($mmol\ h^{-1}\ g^{-1}$)	Scavenger	Light source (Xe lamp)	Ref
**$Cd_{0.9}Co_{0.1}S$**	**8.009**	0.35 M Na_2S-0.25 M Na_2SO_3	UV-visible light	**This**

				work
$CdS/MoS_2/Mo$	4.54	0.35 M Na_2S-0.25 M Na_2SO_3	λ > 420 nm	[71]
Co-NG/CdS	1.382	1.0 M $(NH_4)_2SO_3$	λ > 420 nm	[72]
CdS/MoS_2	1.145	10% TEOA	λ > 400 nm	[73]
CdS/MoS_2	1.36	5% lactic acid	λ > 420 nm	[74]
CdS/Ni-Mo-S	0.838	10% TEOA	UV-visible light	[75]
CdS@CuS	1.654	lactic acid	λ > 420 nm	[76]
Mo_2C/CdS	7.7	20% lactic acid	λ > 420 nm	[77]
CdS/Ti3+/N-TiO2	4.47	0.35 M Na_2S-0.25 M Na_2SO_3	λ > 420 nm	[78]
$ZnIn_2S_4/CdS$	3.07	0.35 M Na_2S-0.25 M Na_2SO	UV-visible light	[79]
$CdS/SrTiO_3$	1.322	10% methanol	UV-visible light	[80]
$CdS/TiO_2(B)$	1.577	0.1 M Na_2S and Na_2SO_3	UV-visible light	[81]

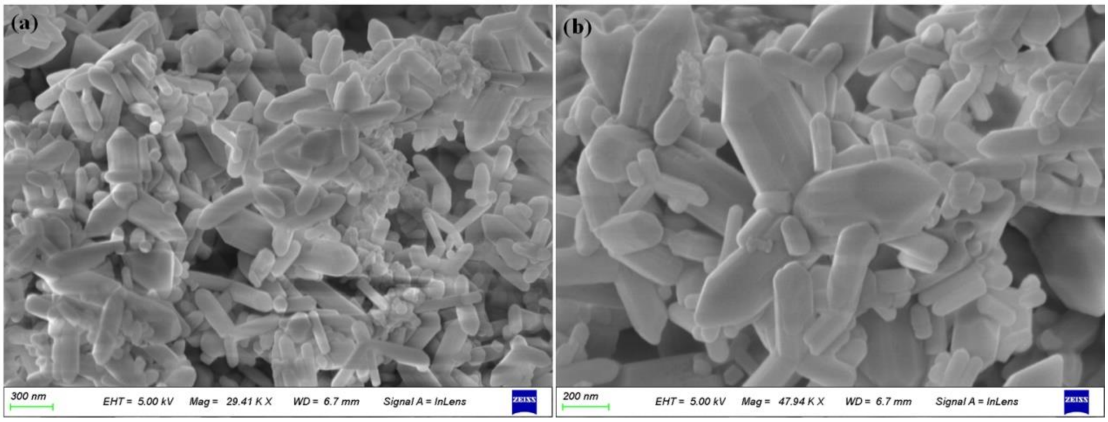

Figure S2 SEM images of $Cd_{0.9}Co_{0.1}S$ NRs after cycle reaction

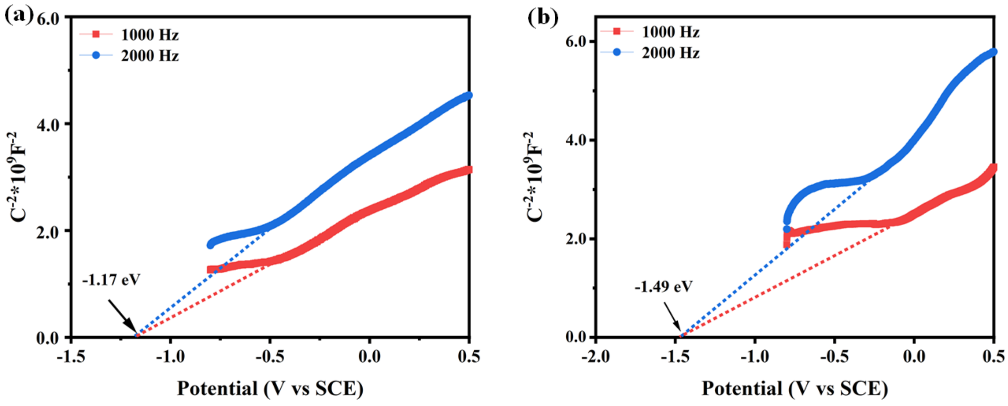

Figure S3 Mott-Schottky plots of pure CdS (a), and $Cd_{0.9}Co_{0.1}S$ NRs (b).

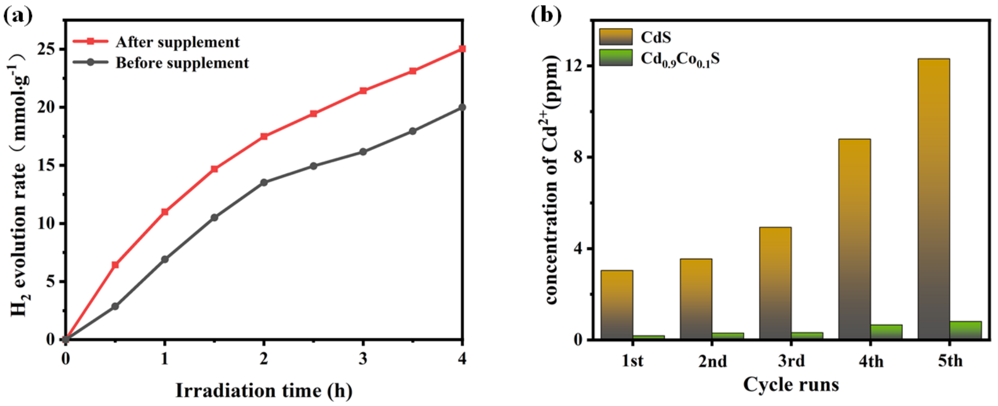

Figure S4 Supplementary sacrificial agent and catalyst after the 5th cycle of the stability test (a), and the Cd^{2+} concentration change in solution of pure CdS and $Cd_{0.9}Co_{0.1}S$ NRs with light irradiation time.

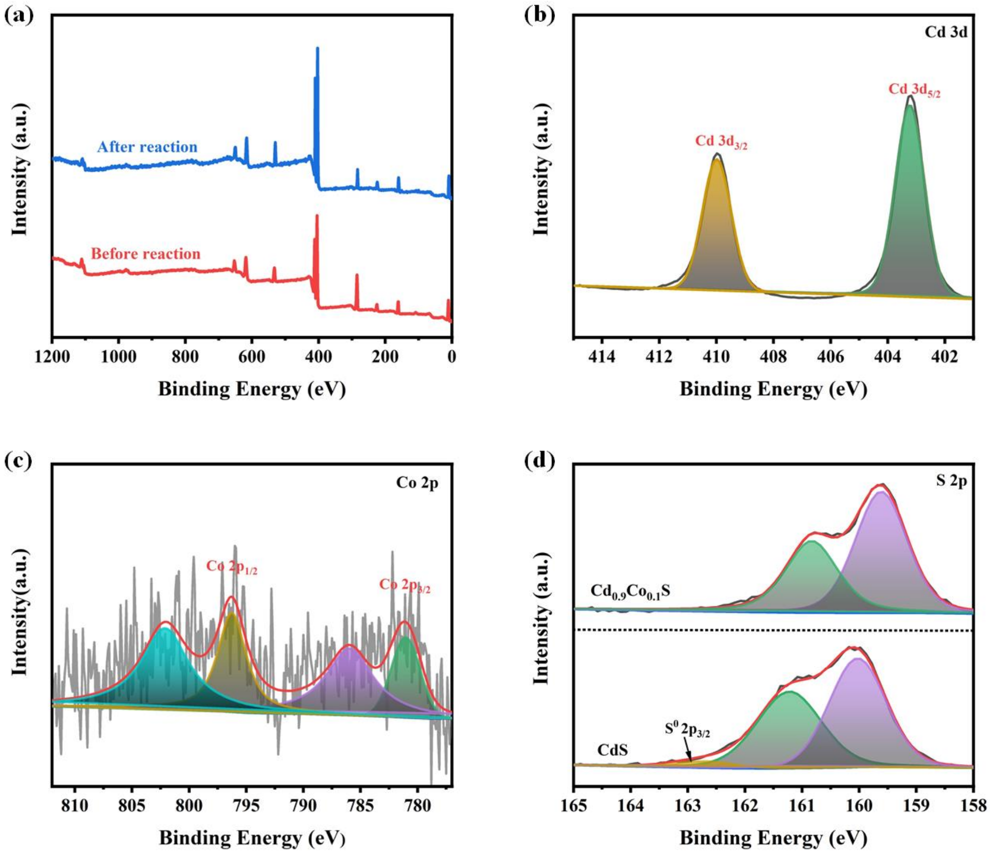

Figure S5 XPS pattern of the CdS and $Cd_{0.9}Co_{0.1}S$ NRs after cycle reaction.

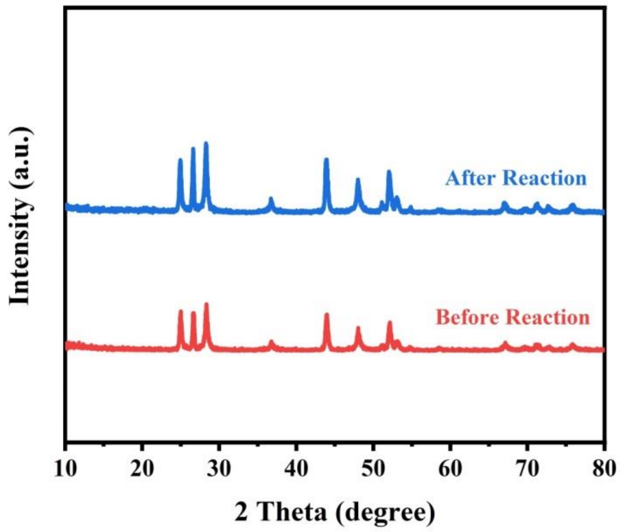

Figure S6 XRD pattern of the $Cd_{0.9}Co_{0.1}S$ NRs before and after cycle reaction.

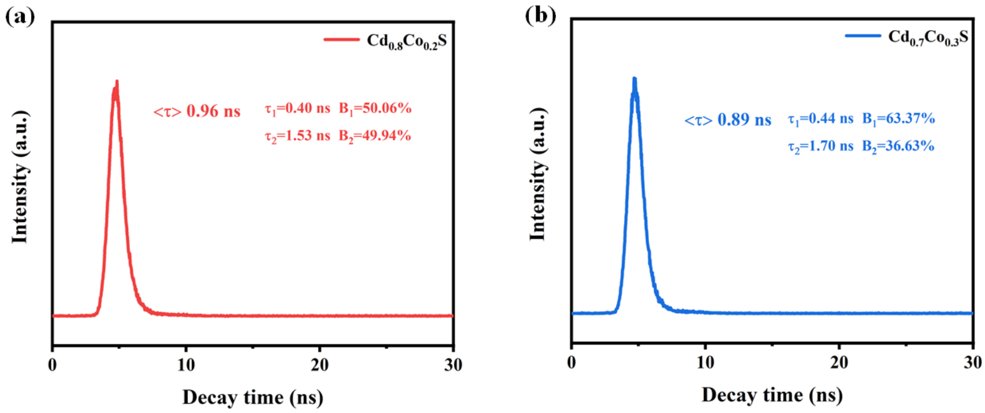

Figure S7 TRPL decay spectra of $Cd_{0.8}Co_{0.2}S$ and $Cd_{0.7}Co_{0.3}S$ NRs.

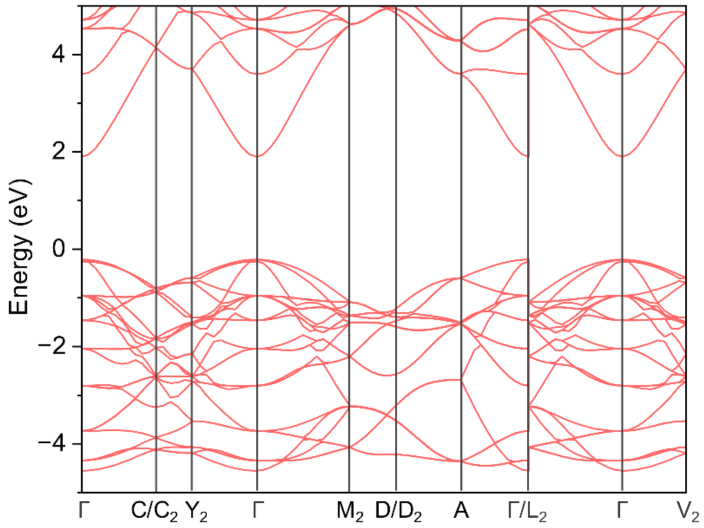

Figure S8 Electronic band structure of CdS.
